# Mental health and routine police work: a systematic scoping review

**DOI:** 10.1186/s40352-026-00405-4

**Published:** 2026-03-27

**Authors:** Martin Webber, Elizabeth Hughes, Tobias Kammersgaard, Charlie Lloyd, Andrew Papworth, Oznur Yardimci

**Affiliations:** 1 Vulnerability and Policing Futures Research Centre, School for Business and Society, University of York, York, UK; 2 School of Health and Wellbeing, University of Worcester, Worcester, UK; 3 Department of Psychology and Behavioural Sciences, Aarhus University, Aarhus, Denmark

**Keywords:** Policing, Mental health, Mental health crisis, Emergency mental health response

## Abstract

**Background:**

There is increasing attention to the amount of contact between police and those experiencing mental health problems. This includes the amount of time and resource spent by police in attending to calls related to mental health, as well as what the role of the police should be in relation to other agencies, including mental health and social care services. The aim of the scoping review was to identify what research has been conducted about police response to mental health in terms of decision making, responses, as well as skills and interventions.

**Methods:**

A scoping review using the PRISMA extension for scoping reviews was conducted. Papers were included if they reported research on routine police work when responding to people with mental health problems or experiencing mental distress. Seven databases were searched and a two-stage searching procedure was followed. Screening and data extraction was completed by two researchers working independently.

**Results:**

A total of 14,608 texts were found; 7,164 were removed as duplicates and 7,281 were removed after screening titles and abstracts. After full text screening a further 100 were excluded, leaving 63 papers for data extraction. About half of the papers included police only as participants (*n* = 31) and only 10 papers were from the perspective of people with lived experience. Three themes emerged from the thematic analysis: “policing decisions and actions”; “coordinating with other providers” and “lived experience”.

**Conclusions:**

Police have skills that have been developed experientially rather than through formal training. There is often a lack of shared understanding between police and other agencies leading to uncertainty about how to best help the person with mental health concerns. Police can and do make a positive contribution to the multi-agency response to people with mental health problems, though should not necessarily be the first (or only) response to mental health calls. Most of the research to date has focused on the police role in mental health crisis. Further research is needed on understanding the police role in encountering mental health problems in routine work (as victims, witnesses or perpetrators).

**Registration:**

The protocol was published on Open Science Framework (tcs45).

## Background

There have been large increases in the incidence of mental health problems, exacerbated by COVID-19, which has particularly affected women, young people and minoritised ethnic groups (Dykxhoorn et al., 2024; Moreno-Agostino et al., 2023; Morris et al., 2025; Proto & Quintana-Domeque, 2021). Most are treated in primary care though a smaller proportion of people who experience more significant and enduring mental health problems including psychosis, bipolar disorder, personality disorder or severe depression receive care in secondary mental health services. Social and economic adversity has contributed to this rising demand (Kirkbride et al., 2024), including living with debt, being out of employment and living in a community characterised by socio-economic deprivation (Morris et al., 2025).

Many correlates of mental health problems are also associated with higher police involvement. For example, substance use, homelessness and suicidal ideation often co-exist (Lee et al., 2017), which are also predictors of higher police involvement (Greer et al., 2022). People from Black British, African and Caribbean groups (particularly young Black men) are disproportionately represented in detentions under mental health legislation in the UK (Barnett et al., 2019), and are more likely to be subject to stop and search by the police (Vomfell & Stewart, 2021). Poverty and racism are both associated with an increased frequency of mental health problems and police involvement, highlighting the compound and intersecting nature of structural determinants.

People with mental health problems encounter the police as witnesses, victims or perpetrators of crime, or due to behavioural disturbances in public places or through a third party being concerned for their welfare. There is a perception among senior police officers that a high proportion of their work is related to ‘mental health calls’ (Osborne, 2022). However, it is difficult to determine the extent to which police officers encounter people with mental health problems in their routine work as there are varying definitions of ‘mental health’ across police forces, and differences in coding and recording in police systems (Frederick et al., 2018). Common terms used by the police which may indicate the presence of a mental health problem include: ‘mentally ill’, ‘mental disturbance’, ‘people in crisis’, ‘people with mental illness’, ‘people with behavioural health challenges’, or ’emotionally disturbed persons’, for example (Frederick et al., 2018). Studies of police databases have found that rarely more than 10% of incidents involved a person with a mental health problem (Kane et al., 2021; Koziarski et al., 2022; Langton et al., 2021; Livingston, 2016).

In England and Wales, the policy ‘Right Care, Right Person’ (Home Office & Department of Health and Social Care, 2024b) has been introduced. This policy addresses concerns that the police are not always the most appropriate or skilled agency to respond to calls involving people with mental health problems. It entails diverting some calls to mental health helplines; declining calls for welfare checks from health and social care agencies; and declining requests to respond to people missing from healthcare facilities unless there is a significant likelihood of serious harm to self or others. Evaluations have found that it saves police officer time, but health and social care agencies articulated some concerns about its implementation (Home Office & Department of Health and Social Care, 2024a; Jefferson et al., 2024).

A whole system approach is required to effectively respond to the diverse needs of people with mental health problems (Crawford, 2024). However, police officers often encounter people experiencing mental distress in their routine work and need to respond appropriately. This may involve making rapid decisions about a situation, sometimes without full information being available. In working with people with mental health problems, this may require de-escalating situations without defaulting to tactics of dominance and containment typically used when addressing criminal behaviour. This work often falls into the ‘gray zone’ of policing (Wood et al., 2017) where the threshold for illegality has not been crossed, or is not clear, leaving the police to navigate situations lacking a clear resolution.

The role of police in responding to calls involving people with mental health problems is an important policy consideration in the UK, but is also one of the most urgent and challenging issues that policing faces globally (Police Executive Research Forum, 2023). In particular, the potential harms of police involvement in mental health crises are well documented and these are unevenly experienced. About 20–30% of people shot by the police in the US were experiencing a mental health crisis at the time of their death (Khan et al., 2024; Saleh et al., 2018; Ward et al., 2025). In addition, non-lethal encounters with the police are associated with poorer mental health for Black Americans in the US (McLeod et al., 2020). The police response to the intersection of poor mental health and Black identity in the US has stoked the Black Lives Matter movement (Jordan et al., 2021) and has led to calls to defund the police and increase investment in mental health services (Cummins, 2023). What these dynamics collectively highlight is that mental health is not only a clinical matter but a deeply social one, and that police interpretations of who is risky or needing care, and whose distress is threatening, are shaped within a wider context in which structural racism plays a central role.

Several reviews have focused on police co-response models with mental health services, which predominantly address mental health crises (e.g. Ghelani et al., 2023; Kane et al., 2018; Park et al., 2021; Parker et al., 2018; Puntis et al., 2018; Rodgers et al., 2019; Shapiro et al., 2015; Winters et al., 2015). However, there remains limited empirical understanding of how police interpret and respond to people with mental health problems during routine interactions, particularly in non-crisis situations. ‘Routine police work’ is here defined as a non-specialist response to mental health problems encountered in the course of a police officer’s work. This excludes the work of officers with specialist mental health training or specific co-response models with paramedics or mental health professionals, for example. Mental health crises may be encountered within routine police work, though the focus is on how officers who do not have specialist training, or are not working in a specific co-response model, respond.

This review aims to explore this gap in knowledge by scoping existing research on police responses to people with mental health problems. It aims to explore research on techniques, models and intervention methods used by the police when they respond to people with mental health problems in the course of their routine work, including how they conceptualise mental health and the impacts thereof on what they do.

## Methods

This review has been structured according to the PRISMA extension for scoping reviews (Tricco et al., 2018). The protocol for the review was published on Open Science Framework.

### Eligibility criteria

Peer-reviewed papers were included in the review if they reported a description or evaluation of the police models, techniques or intervention methods that were used in routine police work when responding to people with mental health problems or experiencing mental distress. Papers were included if they were published in English from 2000 onwards and reported empirical research findings. Reviews, commentaries, PhD theses and letters were excluded. Papers reporting training evaluations were also excluded, as these have been reported elsewhere (e.g. Booth et al., 2017), as were papers which focused on co-response models (where mental health and police work together in a team to respond to mental health calls) as these models are not typical of “routine policing” and have been reviewed elsewhere (Puntis et al., 2018).

### Information sources

Systematic searches were conducted in PubMed, Web of Science, Scopus, MEDLINE, PsycINFO, Social Policy & Practice, and Sociological Abstracts. These databases provided a sufficiently broad coverage of the topic area, including studies in policing and mental health, within the time available for the scoping review. A search of grey literature was not included, but further backwards and forwards citation searching and expert review searches took place. Searches were completed in April 2025.

### Search strategy

The following search terms were used in all databases. It was developed in ScienceDirect and then adapted for use in the other databases searched:

(policing [tiab] OR police [tiab] OR law enforcement [tiab]) AND (“mental health” [tiab] OR “mental illness” [tiab] OR “mentally ill” [tiab] OR “mental disorder“[tiab] OR “psychological disorder“[tiab] OR schizophrenia [tiab] OR psychosis[tiab] OR mental*[tiab])

### Selection of sources

A two-stage screening strategy was adopted. Firstly, titles and abstracts of all identified sources were screened against the eligibility criteria. In the second stage, the full texts of the remaining sources were screened against the eligibility criteria. Two researchers independently screened sources at each stage using Covidence. Disagreements were resolved by discussion, with final decisions made by the study Principal Investigators (EH and MW).

### Data extraction process

Data were extracted from eligible studies using a data extraction form designed for the purpose. Two researchers (OY and TK) independently extracted data from the first five papers for reliability verification. Any differences were resolved through discussion. The two researchers then worked independently to extract data from the remaining papers. The two study Principal Investigators (EH and MW) reviewed the extracted data and resolved any queries which emerged in the process.

### Data items

The following data were extracted from papers: country; study design; sample; and study findings about routine policing and mental health.

### Quality assessment

No quality assessment was undertaken as this is not a requirement for a scoping review (Gough et al., 2012). In particular, this review focused on scoping the breadth of research conducted in this field and the themes emerging from the findings. Methods used in the included papers were captured in the data extraction process and summarised in the results section below.

### Synthesis of results

We developed a coding framework for the synthesis of the findings of the papers included in the review. Firstly, two researchers (OY and TK) reviewed the summaries of findings in Tables 1, 2, 3, 4 and 5 and created a preliminary coding structure that included key thematic areas. To test the framework’s relevance and consistency, each reviewer then independently applied it to the findings of two randomly selected papers. Following this, the reviewers compared their independently generated codes to identify any inconsistencies, gaps, or areas for refinement within the framework. The two Principal Investigators (EH and MW) reviewed and provided feedback to finalise the structure. The data, consisting of summaries of findings from each study, were then extracted and transferred into NVivo, and then coded against the finalised coding framework by OY and TK. The coding framework included three main themes which are reported in the results section below.

**Table 1:** Perspectives of those with lived experience of mental health issues and their carers

Citation	Country	Study design	Sample	Main findings
Watson et al. (2008)	USA	Qualitative interviews	20 people with lived experience who had encountered police in last 12 months	Based on prior experience, participants expected to be treated badly/abused by police. Much focus has been on mental health crisis when in fact people with mental illness often come into contact with police due to their location, rather than their activity
Riley et al. (2011)	UK	Qualitative interviews	16 people detained under s. 136 of the Mental Health Act 1983 and 6 carers	Being detained in cells made them feel like “criminals” and dehumanised. Overriding feeling of disempowerment by detainees and carers
Girard et al. (2014)	France	Observation and qualitative Interviews	Observation of 40 interactions. Focus groups with professionals and people with lived experience	Closer working between police and mental health helps homeless people receive care and reduces use of coercive practices. People were more fearful of psychiatrists than police
Livingston et al. (2014)	Canada	Qualitative interviews	60 people with mental health issues with recent police contact	Majority held positive views. A skilled officer who uses minimal force has an increased likelihood of a positive outcome for all.
Koskela et al. (2016)	UK	Qualitative interviews	81 people with mental health issues reporting crime	Their mental health problems were seen as stigmatising and their reports of crime were often discredited or disbelieved by police
Bradbury et al. (2017)	Australia	Qualitative Interviews	6 people with mental health issues; 4 carers; 2 police, 1 paramedic, 3 mental health nurses	When police use powers of detention when violence is not part of the presentation, it is perceived as harsh. Mental health crisis responses were ad hoc and influenced by the attitudes of emergency services rather than the situation
Bendelow et al. (2019)	UK	Interviews, observation, routine data	Data on all s.136 detentions in 2012. 37 interviews with people who had been detained. 250 h observations	S.136 was viewed as lifesaving but also traumatic. Police decisions to use s.136 seems to be appropriate, risk adverse response to distress and suicide prevention especially out of hours (especially when no other service is available).
Jones and Thomas (2019)	Australia	Online survey	People with mental health issues in contact with police	Contextual and situational factors alter individuals’ response to interactions with police. Prior contact with police can impact on current attitudes to police
Baker and Pillinger (2020)	USA	Qualitative Interviews	Carers whose loved one had died after police contact	Police have become the *de facto* response to healthcare issues
Hirschi (2022)	USA	Qualitative Interviews	5 family members of someone living with mental illness who had been involved with the police.	Police have a positive impact and help provide access to services. Police also seen as a barrier when they do not work collaboratively with parent carers.

**Table 2:** Perspectives of police

Citation	Country	Study design	Sample	Main findings
LaGrange (2003)	USA	Semi-structured interviews	150 police officers	University educated officers were more likely to divert to mental health care than officers with other educational backgrounds
Ruiz and Miller (2004)	USA	Survey	164 police departments	When a single officer is despatched to scene (as opposed to 2–3 officers deployed) then they are more likely to take time to de-escalate rather than rush to use force. Half of police feel threatened attending mental health calls. This perception of danger may influence decision to use force quickly
Novak and Engel (2005)	USA	Observations	236 observations with beat officers, 206 COP officers	People with known mental health issues are less likely to be arrested and more likely to be diverted to healthcare. Disrespectful and hostile behaviour is associated with arrest irrespective of mental illness as an underlying factor or not
Sellers et al. (2005)	USA	Survey	182 responses from 3 police departments	Despite not having a specialised response to mental health, the perceived effectiveness of the strategy used by the three police departments is similar to that of other more specialised mental health response programmes
Mclean and Marshall (2010)	UK	Qualitative Interviews	9 police officers	The police officers reported positive relationships with people with mental health problems, though were frustrated by minimal support from other professionals and gaps in mental health service provision.
Godfredson et al. (2011)	Australia	Survey	3,534 police officers	Decision making was influenced by differential weight placed by officers on different sources of information. Police want more training on skills to use when encountering emotional distress.
Erdner and Piskator (2013)	Sweden	Interviews	Police officers	Experience of detaining people and transporting to mental health care was a varied experience. Police noted feelings of uncertainty and lack of control. Lacked theoretical knowledge of mental illness but had practical knowledge gained experientially.
Ogloff et al. (2013)	Australia	Interviews and survey	25 interviews; survey 3534 officers	Understanding of mental health more likely based on experiential learning or personal experience. Police generally feel unsupported by mental health services. Concerns about how much time spent resolving mental health incidents and seen as representing a “drain” on police resources.
Martin and Thomas (2014)	Australia	Interviews	25 Police Officers	Officers were asked about responding to absconding from mental health services. They saw this as a regular event and felt mental health services abdicated responsibility too soon. Information received was often poor, and police were not informed when person was found.
Menkes and Bendelow (2014)	UK	Focus groups	Police officers	Police attitudes to applying s.136 of Mental Health Act in cases of threatening suicide or engaging in self-harm. Use of s.136 was influenced by availability of support and when police see themselves as last resort. Police interpret s.136 as a suicide prevention strategy.
Watson et al. (2014)	USA	Interviews using NOSI	147 police officers	How officers perceived scenarios was based on perceived threat/resistance and substance use rather than influenced by prior training
Martin and Thomas (2015)	Australia	Interviews	25 Police Officers	Police identified people with personality disorder (PD) and expressed frustration and powerlessness when they tried to refer this group to mental health. Emergency Departments were reluctant to assess people with PD, or they were quickly discharged. People with PD were reported to take up a lot of police time especially when mental health professionals said there was nothing they could offer and police were left without any options.
Oxburgh et al. (2016)	UK	Survey	35 Police Officers	Police perceptions and experiences influence interview practice; closed questioning is seen as most effective when actually using more open-ended questioning would be better for those with mental health issues.
Schulenberg (2016)	Canada	Observation of police	74 ride-alongs (637 h)	Officers recognise that criminalisation of mental illness exacerbates situations. They advocate for diversion away from criminal justice system, however may have limited choices and may have to use powers of arrest for public disturbance or minor offences.
Leese and Russell (2017)	UK	Semi structured interviews	Police officers	University educated officers more likely to make a psychiatric referral rather than arrest, than officers with other educational backgrounds
Watson and Wood (2017)	USA	Descriptive data from mental health calls and interviews	Data from 300 officers related to 428 mental health calls 21 interviews with police officers	Every day policing includes a gamut of interventions, least of all, arrest. Police used informal and creative strategies to resolve many calls at scene. Skills include persuasion and de-escalation using information they may have gathered over time related to the individual.
Wood et al. (2017)	USA	Observations of police work	31 ride-alongs with 53 officers; 120 h	Police navigate mental health calls and offer only provisional solutions. This is not through lack of commitment by police, but rather an absence of family and community resources that can address conditions that lead to vulnerability and mitigate crisis.
Bohrman et al. (2018)	USA	Semi structured interviews	15 Police officers	Assessments at scene made by using a combination of information from despatch, collateral contacts and behavioural observations. Neighbourhood context influenced assessment. There is a need to improve quality of information used to inform decisions.
Geijsen et al. (2018)	Nether-lands	Survey	Police and suspects	Psychological vulnerabilities prevalent among suspects. Prevalence significantly under-estimated by detectives and therefore less likely to take these vulnerabilities into account
Lane (2019)	UK	Discourse analysis	Analysis of threads online police discussion forum	There is a resistance to the amount of mental health work in policing and difficulties defining police responsibilities in this area.
Soares and Pinto da Costa (2019)	Portugal	Semi structured interviews	10 police officers	Police felt it was not their responsibility to deal with mental health crises. The time spent on this was their biggest concern. They want increased support from mental health providers, and for the process to be more efficient.
Williams et al. (2017)	UK	Semi structured interviews	82 police including custody, PACE, detention officers	Current practices in custody suite are not sufficient for the safety of detainees and officers. Limited training, reduced resources and reliance on experiential knowledge and a non-standardised approach to provision of health care professionals in this area is contributing to the risk for potential harm.
Marsden et al. (2020)	UK	Semi structured interviews	15 police officers	Officers had mixed feelings about involvement in mental healthcare. They identify situations where they are necessary, however do not feel equipped to manage mental health emergencies
Watson et al. (2021)	USA	Analysis of reports and survey	428 officer reports	Calls that were pre-identified as “mental health” more likely to be linked to mental health services, less likely to be arrested. Call location was important: officers used their knowledge of locations to inform decisions. The role of call handlers and despatchers in shaping police response requires more investigation.
Wittmann et al. (2021)	Germany	Survey	958 Police Officers	Majority experienced conflictual interactions with people with mental illness multiple times a month. One third reported fear during these interactions. Less knowledge about mental illness was correlated with more anxiety.
Wood et al. (2021)	USA	Observations	Data collected from 36 ride-alongs	Officers responded to a variety of mental health-related calls. Common theme was “going off meds” combined with other situational factors. Police also felt that issues in the broader health and social care system constrained their ability to effect positive outcomes.
Gillooly (2022)	USA	Observation and call for service data	Observations of 31 call handlers Analysis of 20,764 call for service data	Variation in how 911 call handlers code mental health calls. This impacts on how police then respond and code incidents on scene. There is a tendency to perceive incident as more severe due to initial call handler assessment.
Miles-Johnson and Morgan (2022)	Australia	Semi structured interviews	25 general duties police officers	Mental health crisis is underpinned by trial-and-error practices as officers are insufficiently trained. Police are resistant to accept tasks perceived to be “welfare work”. They saw policing of people with mental illness as core role, but some did not see it as part of policing and the responsibility should lie with other agencies.
Cohen (2023)	USA	Interviews	57 police officers	Officers felt unequipped to deal with mental health due to lack of training and also don’t perceive this work as part of police role.
Pifer (2023)	USA	Observation data	120 h in the field, and notes with 84 police officers	Examination of hybridization of policing and mental health, police hold on to policing roles even when expectation to incorporate mental health work is present.
Shefner et al. (2023)	USA	Focus groups	56 police officers	The police felt police involvement in mental health was inappropriate but significant barriers to de-centring police. They recognised their presence can intensify fear and hostility. They valued CRT training but not always clear how to use it in routine practice.

**Table 3:** Papers using administrative data only

Citation	Country	Study design	Sample	Main findings
Steadman et al. (2000)	USA	Analysis of despatch calls	100 police despatch calls for “emotionally disturbed persons”	Large variation across sites in proportion of calls that resulted in specialised response. One difference was the availability of a drop-off centre for people with mental illness (which had a no refusal policy). Collaborations between criminal justice and mental health systems could reduce inappropriate use of jails for people in mental distress.
van den Brink et al. (2012)	Nether-lands	Analysis of police records	Calls relating to mental health crisis over 1 year	Substantial number of calls related to crisis. Half of the people were out of contact with mental health services at the time. Police play a pivotal role in connecting them to mental health services.
Charette et al. (2014)	Canada	Retrospective analysis police records	6128 records of police records on 3 randomly selected 4 days in 2006	Intervention for minor offences were more likely to lead to arrest if the person has mental health problems. Police intervention with mental health used 87% more resource than those without mental health issues; however, this higher resource use reflects the more complex needs of the population.
Holman et al. (2018)	New Zealand	Data forms relating to police contact	86 forms collected anonymised data from mental health incidents	Maori were over-represented in police responses. Many did not require mental health admission raising questions about the need for and the nature of police involvement in these cases. Non-admission to hospital may indicate that unnecessary force may have been used to detain and transport for mental health assessment.
Shore and Lavoie (2019)	Canada	Official police data	Calls primarily identified as mental health related *n* = 400	Police encounters with mental health were often with young people and those of racialised minorities. Half were resolved by police making mental health act detentions and half of these led to hospital admissions. Things that increased detentions include self-harm, abrupt cessation of medication, and third-party concerns (public, paramedics etc.) Police made mental health referrals in 40% of non-detained cases.
Hendy et al. (2022)	New Zealand	Content Analysis	Calls identified as mental health between 2010–2016	Significant increase in calls to police related to mental health; many were not emergency or related to an offence; many were initiated by person experiencing mental health issues. Possible link to reduction in mental health service resource.
Mitchell et al. (2022)	USA	Analysis of Call for Service calls (CFS)	60,593 calls	Contextual ambiguity and issues with data completeness leads to mis-classification of mental health calls. Use of force may be due to misclassification of calls and not down to police bias.

**Table 4:** Papers including police and partner agencies

Citation	Country	Study design	Sample	Main Findings
Saunders and Marchik (2007)	USA	Service evaluation using focus groups	11 law enforcement, 10 service providers, 7 programme developers	During the process of establishing a jail diversion programme, diverse perspectives from police and care providers lead to refined shared goals. Feedback indicated officers wanted improved communication with programme providers and more opportunities for training.
Wood and Beierschmitt (2014)	USA	Spatial analysis of trans-portations, qualitative interviews and focus groups	Police, outreach workers and partner agencies	Promotes adoption of enhanced upstream intervention with twin pillars of targeted case management and targeted place management, in order to break the cycle of repeat crisis interventions. Police have role to play and can be used more effectively if their role is more focused.
Genziani et al. (2020)	UK	Qualitative interviews	4 police officers and 3 ambulance workers	Police felt use of s.136 Mental Health Act sometimes felt like a betrayal. They felt under pressure to prioritise other emergencies over mental health crisis. There is friction between emergency services regarding responsibilities of care.
van Steden (2020)	Nether-lands	Qualitative using interviews and observations	Policy makers, police, and district nurses	Community police and nurses had developed an informal team play however the multi-agency network each with its own bureaucratic methods tends to clash and undermine the informal team play on the streets.
Krayer et al. (2018)	UK	Interviews and focus groups	37 interviews and two focus groups (*n* = 7 and 9) with police, mental health and social care staff	People who required a lot of police time due to antisocial behaviour tended to have complex mental health issues. Some mental health staff felt police were too quick to resort to control. They also felt that police regarded any form of distress as mental health problems and call for mental health assessments unnecessarily.
Wondemaghen (2021)	UK	Qualitative interviews	30 police officers and mental health nurses	Use of mental health street triage had alleviated the previous issues of police being overwhelmed with mental health crisis and use of police cells. Working alongside mental health helped with shared decision making and responsibility and ensured police cells were avoided.
Daggenvoorde et al. (2022)	Nether-lands	Qualitative interviews	8 police officers and 8 members of mobile crisis team	Police feel they are not well trained in mental health and can inadvertently escalate situations. The mobile crisis team try to prevent hospitalisation as they see people recover better in their own environment, yet police see hospital admission as a good outcome as they perceive that is where they will be safe and get help.
Kuehl et al. (2023)	New Zealand	Survey	57 police officers, 29 paramedics, 33 mental health professionals	Current single agency model is not working. Staff found limited mental health training, poor interagency referrals and difficulties accessing support from mental health services.
Pope et al. (2023)	USA	Qualitative interviews and focus groups	94 stakeholders	Decisions on how, when and where to intervene are influenced by competing demands of stakeholder agencies.

**Table 5:** Papers that refer to Crisis Intervention Training (CIT) and impact on policing

Citation	Country	Study Design	Sample	Main Findings
Canada et al. (2010)	USA	Qualitative interviews	20 police officers working across 2 high CIT and 2 low CIT districts	Irrespective of whether they had received CIT training, all perceived the benefits of CIT implementation in their district. CIT trained officers use “softer” skills when responding to mental health and divert rather than arrest
Watson et al. (2010)	USA	Interviews and surveys	112 police officers across 4 districts	CIT trained officers more likely to divert to health than arrest. Moderators to this include level of resistance and presence of a weapon
(Ritter et al., 2011)	USA	Analysis of reports	2174 CIT trained officer reports	Dispatcher classification of a call has an influence on officer decisions at scene and therefore dispatchers need to be included in CIT training. CIT trained officers more likely to divert to health care than arrest or left at scene.
Canada et al. (2012)	USA	Interviews	20 police officers	CIT-trained officers have the ability to resolve mental health calls with less force, and more likely to link people to appropriate services rather than arrest.
Kubiak et al. (2017)	USA	Interviews and administrative data	79 police officers and call data	CIT requires key law enforcement and mental health stakeholders to be in continuous dialogue to establish and maintain the programme in order to integrate differing perspectives and problem solve.
Bratina et al. (2020)	USA	Analysis of administrative data	405 documented CIT encounters	CIT-trained officers may be more likely to hold less stigmatising attitudes; more likely to understand mental health needs, and more likely to divert from criminal justice to health.

## Results

### Selection of sources

**Fig. 1:**
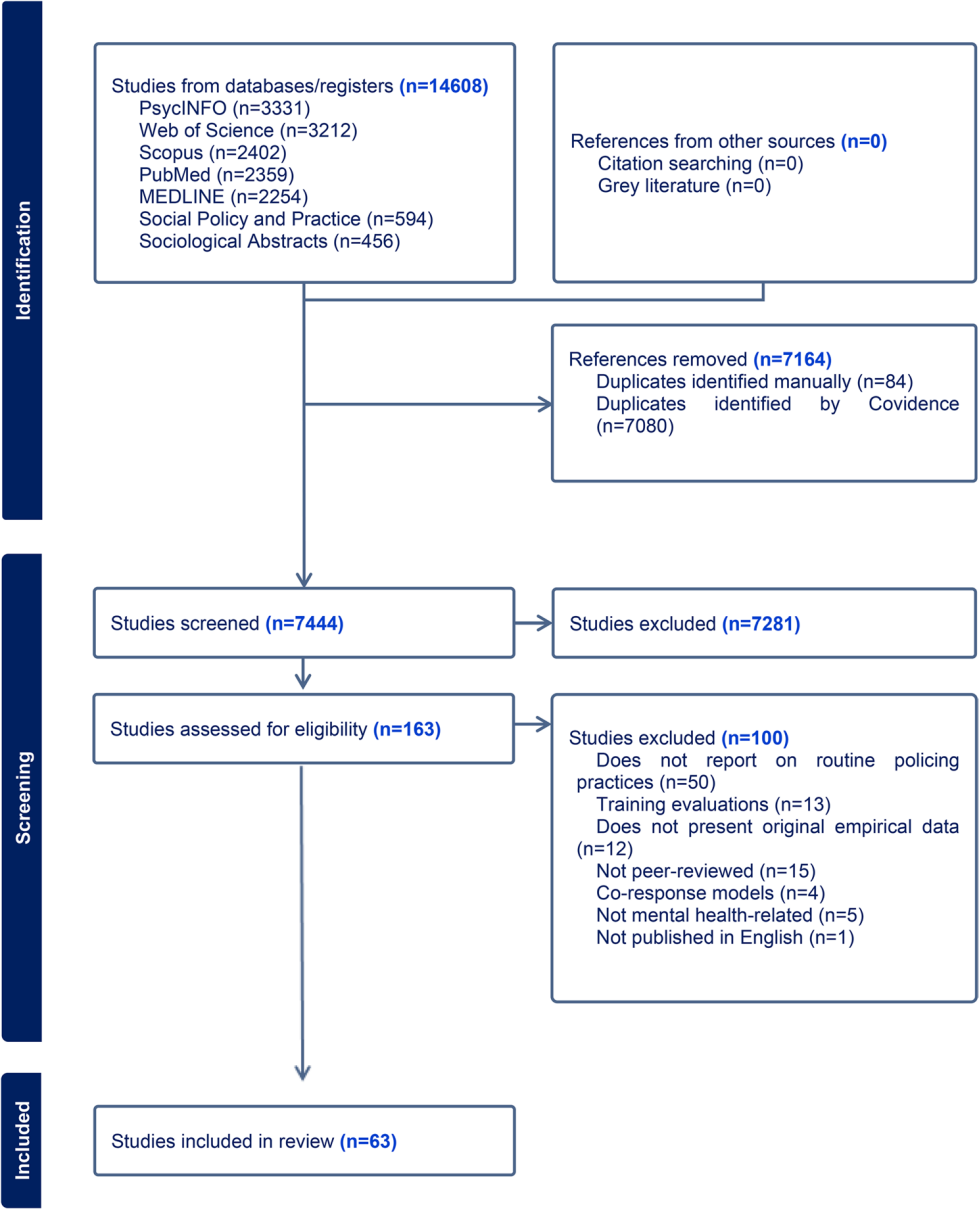
PRISMA Flow Diagram

Searches identified 14,608 potentially eligible studies, which reduced to 7,444 after duplicates were removed. 7,281 sources were removed in the title and abstract screen, and a further 100 were excluded in the full text screen. Sixty-three studies met the criteria for inclusion in the review (Fig. 1).

### Characteristics of sources

Of the 63 papers included, only ten were focused on the lived experience of people with mental health conditions and/or their carers (Table 1). About half of the included papers included police participants only (*n* = 31) (Table 2), which included a mix of qualitive interviews, observations of practice and surveys. An additional seven papers included analysis of police administrative data (Table 3). Nine papers included police and partner agency participants (such as social workers, mental health staff and paramedics) (Table 4). These met the criteria for ‘routine policing’ as they did not focus on co-response models. Finally, six papers focused on Crisis Intervention Training (CIT), but we only included papers that reported data on the impact of CIT on routine policing (Table 5).

The papers were methodologically diverse and, although we did not conduct a formal quality assessment, it was apparent that they varied in methodological rigour. For example, three of the 35 papers which analysed qualitative data from semi-structured interviews had samples of fewer than ten participants (Genziani et al., 2020; Hirschi, 2022; Mclean & Marshall, 2010), and others were only slightly larger (e.g. Bohrman et al., 2018; Bradbury et al., 2017; Marsden et al., 2020; Soares & Pinto da Costa, 2019). Although sample size is not the only determinant of methodological quality in qualitative studies, it can restrict the depth and breadth of conclusions which can be drawn from the data. Similarly, the response rates of the surveys reported in the included studies varied from 17% (Sellers et al., 2005) to 93% (Godfredson et al., 2011), with a concomitant large range in sample sizes, indicating that more weight should be given to some studies than others.

### Synthesis of results

The data from the review was synthesised into three main themes with sub-themes within each one.

#### Policing decisions and actions

##### Interpersonal skills

There was consensus across several papers that interpersonal skills were important in interactions with people with mental health problems. For example, a small study of parent carers interviewed in the US reported that police behaviour with people with mental health problems can make a real difference to perceived outcomes (Hirschi, 2022). The participants in the study called for the police to take time to listen to carers and work with them to meet their loved one’s needs (Hirschi, 2022). It was evident, however, that police officers were not always sensitive to the additional needs of people with mental health problems. In addition, research conducted in the Netherlands suggested that detectives did not adapt their interrogation approach when responding to people with mental health problems (Geijsen et al., 2018).

##### Time constraints as barriers to use of ‘soft skills’

Time pressures often prevented police from improving the way they responded to people with mental health problems (Genziani et al., 2020; Oxburgh et al., 2016; Soares & Pinto da Costa, 2019). For example, police detectives were often concerned that the “custody clock was ticking” and that some of the adaptations required to accommodate for someone’s needs could “slow them down”. Oxburgh et al. (2016) recommended that police practices during interrogation should be tailored to account for mental health (such as regular breaks, taking time to explain concepts) as well as obtaining a Forensic Medical Examiner assessment of “fitness to interview” and/or use of appropriate adult during the interview. In a small UK study of police detention powers under the Mental Health Act 1983, Genziani et al. (2020) highlighted the tension between the time required to use interpersonal skills to engage with someone in order to gain their trust and de-escalate the situation on the one hand, and the use of Mental Health Act 1983 powers to resolve an issue quickly on the other. Another barrier highlighted in a US study which used police ride-alongs was that much of the police contact with people with mental health problems was “reactive”, creating pressures for the police to respond to the needs of people they do not know in situations that are unfamiliar to them (Wood et al., 2021).

##### Training

Several scholars highlighted the need for improved interpersonal skills training for the police. Miles-Johnson and Morgan (2022) called for officers with specialist mental health intervention skills who had a clear understanding of mental health crises and complexity of mental health issues. Police officers themselves recognised the need for further training, which was captured in a large Australian survey which found that they wanted more input in how to approach, engage and reassure a distressed person (Godfredson et al., 2011).

Kubiak et al. (2017) advocated for the types of skills taught in Crisis Intervention Team (CIT) training, which includes focusing on de-escalation, the use of open-ended questions, mirroring responses and the importance of body language in interpersonal skills. Canada at al. (2012) reported that after CIT training police were more likely to divert people with mental health needs to care rather than arrest. In addition, the training had given them new skills in communication and de-escalation that they had not learnt “on the job”.

Wood et al. (2017) argued that there are a lot of untapped skills and knowledge already held by police officers in the US with regards to the people that they encounter and that this could be deployed in a different way. They found that the relationships the police had formed with particular individuals (through regular contact in neighbourhood-based policing) enabled them to have a broader understanding of their histories and behavioural patterns, and that this provided officers with an understanding of how best to secure compliance and resolve situations (Wood et al., 2017).

#### Coordinating responses with other service providers

##### Timely access to mental health services

Availability of mental health services, especially out of hours (i.e. evenings and weekends), was a significant concern for the police. A small UK study found that police attributed an increase in contact with people with mental health concerns as a failure of the mental health system (Mclean & Marshall, 2010). Often, the police found themselves waiting a long time for mental health services to respond to a request for advice or referral, or for a formal assessment for detention under mental health law (Kuehl et al., 2023). These delays were seen as “unproductive time” (Kuehl et al., 2023) or “wasted time” (Daggenvoorde et al., 2022).

The police were often concerned that a delayed or absent response from mental health services would inevitably lead to adverse outcomes (such as self-harm or interpersonal violence). Police in this situation often felt they were left with no choice but to use their powers of arrest or detention under mental health law as their only way of resolving the situation (Daggenvoorde et al., 2022; Kuehl et al., 2023; Mclean & Marshall, 2010; Menkes & Bendelow, 2014). Menkes and Bendelow (2014) reported that police in England and Wales perceived the use of police detention powers under s.136 of the Mental Health Act 1983 as a “suicide prevention strategy”. Bendelow et al. (2019) reviewed s.136 data from 2012 and found that it was used in response to suicide or self-harm on 80% occasions and that most occurrences took place out of hours when mental health services were less likely to be available.

Supporting these findings, Schulenberg (2016) found that limited community mental health resources, particularly after-hours, led to officers with few options other than to take no further action or to use their legal powers. Similarly, Kuehl et al. (2023) reported that police in New Zealand viewed their only options for people with emotional distress were either police cells or emergency departments, but they also recognised that this was “humiliating or traumatising”. Custody officers in the UK expressed frustration that vulnerable people ended up in custody for low-level crime, when this could have been avoided with improved mental health services in the community (Leese & Russell, 2017).

Daggenvoorde et al. (2022) reported that police in The Netherlands viewed a hospital admission (even if involuntary) as a good outcome as it gave them ‘peace of mind’ that the person was now safe, and no longer their responsibility. Miles-Johnson and Morgan (2022) also reported that Australian officers felt a sense of relief when a response to a mental health crisis included mental health practitioners. However, Ogloff et al. (2013) reported that Australian police felt unappreciated by mental health colleagues and, conversely, some mental professionals in a UK-based study perceived that the police wanted to resort to controlling tactics too readily (Krayer et al., 2018).

##### Different thresholds for intervention

The priorities of police officers and mental health professionals are different as regards mental ‘distress’ and mental ‘illness’. For instance, Menkes and Bendelow (2014) found that the police responded to situations where there was significant emotional distress, though, in contrast, mental health services were focused on the assessment and diagnosis of mental ‘illness’, and were less concerned about the associated ‘social disturbance’ that the police must respond to. Another example of where there were differences between the police and mental health professionals was with people who had a dual diagnosis of both a mental health condition and a substance use disorder. Menkes and Bendelow (2014) reported that co-existing drugs and or alcohol use were an exclusion from s.136 ‘place of safety’ suites, leaving the police with only the custody suite as an option, which they saw as a ‘last resort’. Mental health professionals perceived police as interpreting any signs of distress as mental health problems and that many requests for assessments were unnecessary (Krayer et al., 2018). These findings indicate a difference between agencies about thresholds and remit but also suggest there may be a gap in provision for people who may not meet criteria for mental health services, yet experience mental distress that requires some intervention.

##### Relationships and working together

Genziani et al. (2020) found tensions between paramedics and police in London in terms of roles and responsibilities around emergency detention under s. 136 of the Mental Health Act 1983. Miles-Johnson and Morgan’s (2022) study in Australia found that police officers felt they could not be a viable alternative to mental health services given the authoritarian nature of their role. However, Girard et al. (2014) reported on a partnership between homeless outreach workers and police in France, and found that despite differences in culture, training, and practice, the two parties were able to work collaboratively. This required both parties being able to extend themselves beyond their traditional roles to develop common strategies and interventions. This in turn enabled people to receive appropriate care, and to reduce coercive practices, including involuntary hospitalisation and arrest.

The included studies highlighted several ways to improve partnerships and collaborations. For example, Sellers et al. (2005) described an initiative in Newark, New Jersey, which established a multi-agency advisory board designed to integrate the different perspectives of police officers and mental health professionals. This led to patrol officers working effectively with local emergency responders and hospitals, which was associated with a reduction in arrests for people with mental health problems. This aligned with Watson and Wood’s (2017) recommendation for increased and direct access to mental health care that is less coercive than emergency psychiatric facilities.

Other studies also support the need for alternative crisis options (Mclean & Marshall, 2010). Kubiak et al. (2017) described a crisis centre in the UK that had a ‘no refusal’ policy, which meant the police could leave the person there and quickly return to duties. In a study of UK custody officers (Leese & Russell, 2017), there was a consensus that having a mental health nurse working in custody would be beneficial, enabling the early identification of mental health problems.

#### Lived experience perspectives

Of the 63 papers included in this review, only ten papers focused on the experiences of people with mental health problems and/or carers when they encountered the police, and these studies were among the smallest included in the review.

##### General perspectives on the police

Livingston et al. (2014) undertook 60 interviews with people with mental health problems in Canada in relation to their most recent police encounter. Police interactions were common, mostly unrelated to crime, and resolved without arrest. Although there were some experiences of the police using force, many of the participants held positive attitudes towards the police and were generally supportive of the idea of information sharing between agencies that would allow the police to have access to mental health-related information. In contrast, a study in the US found that participants with mental health problems generally distrusted police and expected to be treated badly by them (Watson et al., 2008). Due to low expectations, they considered ‘not being abused’ as a good outcome, even if police behaved unkindly or did not listen to them. Watson et al. (2008) concluded that fairness and respect can affect outcomes of police encounters with people with mental health problems, and that procedurally-just treatment could potentially improve outcomes for all. Similarly, Jones and Thomas (2019) found that the procedural elements of police encounters and prior contacts with the police in Australia were important predictors of cooperation from people with mental health problems. As a result, they emphasised the need for police to be mindful of their interactions and behaviours during all encounters. The participants in one study even suggested rewarding the police for positive practices and increasing the opportunities for positive interpersonal contact between people with mental health problems and the police (Livingston et al., 2014).

A study in England found that when people with mental health problems reported a crime they said that they were often discredited, disbelieved or even blamed by police because of their mental health (Koskela et al., 2016). The experience of detention in a police cell was described by people with mental health problems as a distressing and stigmatising experience (Bendelow et al., 2019). A small study found that detention was perceived as an unusually harsh treatment when used with people who were not being violent, even if the police officers were acting in a kind and gentle manner (Bradbury et al., 2017). People with mental health problems objected to being detained in a police cell, because they felt they were being viewed as a criminal, even though they had not committed a crime (Riley et al., 2011), with many describing it as stigmatising and distressing (Bendelow et al., 2019). However, a more recent study found that only three out of 37 participants found that the use of police mental health powers (s.136 Mental Health Act 1983) against them had been used inappropriately (Bendelow et al., 2019). Though it is interesting to note that a small number of interviewees in this study preferred being detained in police custody, than being detained in a s.136 suite, as police custody gave them a sense of safety, illustrating how preferences can vary.

##### People with mental health problems as victims

The studies included very little data on the experiences of people with mental health problems as victims of crime. A UK study explored their experiences of reporting crime to the police and found mixed experiences, though negative experiences were more common, which had detrimental effects on the participants’ emotional well-being and mental health (Koskela et al., 2016). Some of these negative experiences related to wider problems, such as a lack of resources in the police to pursue certain crimes. However, the labelling and stigmatising attitudes of officers may contribute to negative experiences, such as dismissing, disbelieving or blaming victims with mental health problems (Koskela et al., 2016). Furthermore, the study suggested that future research could explore police experiences and perceptions of victims with mental health problems.

##### Police vs. other service providers

Some studies found that when participants compared the police with other service providers, they often reported more positive experience of police officers than some mental health professionals. For example, Bendelow et al. (2019) reported that a female participant described the police as more likely to take their distress seriously than mental health professionals, and to treat them with more kindness and compassion. They described mental health professionals as treating them as a ‘nuisance’ and ‘seeing them as a diagnosis rather than as a person’ (Bendelow et al., 2019). Similarly, some participants of a study of experiences of homeless people with mental health problems in France described the police as sometimes using less coercion than mental health outreach teams, and that the participants were more fearful of psychiatrists than the police (Girard et al., 2014).

##### Carers' perspectives

Studies which have focused on the perspectives of carers of people with mental health problems have found both positive and negative experiences of the police. A small US-based study found that carers expressed gratitude towards the police for responding to calls when their loved ones were in crisis, and when they helped them provide access to services that they had not succeeded in obtaining themselves (Hirschi, 2022). This study also found that police could be seen as a barrier when they did not take the time to listen to carers or work with them to meet the needs of people with mental health problems (Hirschi, 2022). An Australian-based study found that carers reported that individual police officers respond in different ways, with the best experiences being those when the police responded with patience and empathy and the worst related to when police officers had poor mental health expertise (Brennan et al., 2016).

Carers reported difficulties in accessing services, both prior to and after involuntary interventions; that they experienced delays in service provision; that they felt services had resourcing issues; and that they believed that there were issues with interagency communication and coordination (Bradbury et al., 2017; Brennan et al., 2016). One study highlighted how disempowerment was an overriding feeling among carers whose loved ones had been detained (Riley et al., 2011). Another discussed the trauma that carers sometimes felt after witnessing a loved one being detained by the police and the guilt they felt when they had been the one who had initially involved the police and then something had gone wrong (Brennan et al., 2016). Baker and Pillinger (2020) examined 43 police-related deaths in the US by interviewing the remaining close relatives. They found that some carers expressed concerns that, in the case of the deaths of their relatives, the police had been called when mental health services or an ambulance would have been more appropriate. In some cases, carers equated the 911 call with the death of their loved one: they presumed the police response would result in a confrontation (Baker & Pillinger, 2020).

## Discussion

### Summary of results

This synthesis of findings from studies of non-specialist police responses to mental health problems has found that many police officers use their interpersonal skills to resolve crises, though feel constrained by time or a lack of training. They often experience frustration with a lack of co-response options and difficulties in obtaining a timely response from mental health services. Police officers’ interface with other services was occasionally challenging and outcomes for people with mental health problems encountering the police were not always optimal. Many people experienced police detention as criminalising their mental health, though some reported positive experiences. Although the review included a relatively large number of studies, they were predominantly conducted from the perspective of the police and were often descriptive in nature. However, they revealed that routine policing rarely took a whole system approach to responding to people with mental health problems (Crawford, 2024).

### Contribution to the literature

This review has uniquely focused on the ‘routine policing’ of people with mental health problems. It has found that there has been much less research on the views of people with mental health problems and their carers than there has been from the perspective of police officers and health professionals. This means that we do not yet have a full understanding of the nature of policing of people with mental health problems. Equally, most of the included studies focused on police responses to mental health emergencies, including when they are called to people attempting suicide or are creating a public disturbance because of a mental health crisis. A limited focus on ‘everyday policing’ means that research in this field has been concentrated on ‘crisis’ policing and its seemingly close relationship to violence and incidents concerning heightened risk. People with mental health problems are rarely viewed as witnesses or victims in the literature. This is in contrast to reality, in the UK at least, where most contact with people with mental health problems is in routine policing or their mental health does not feature as a presenting issue.

This narrow focus in the literature needs attention as it overlooks how wider structural factors shape who comes into contact with the police and how they are responded to. Institutional racism plays a significant role in the over-representation of people from Black ethnic groups in England and Wales in stop and searches, arrests or detentions under police mental health powers in contrast to white ethnic groups (Home Office, 2024). Contextual factors which compound this, such as the individual’s socio-economic background, the type of neighbourhood they live in or the context of the particular encounter, are also shaped by institutional racism. Disproportionate outcomes and experiences undermine trust in the police and raise concerns about procedural justice and stigma, which emerged in this review as being important dimensions of the police response to people with mental health problems. If people perceive their treatment by police as fair and respectful, they are likely to experience less stress and anxiety than those who perceive their treatment as unfair or discriminatory. Conversely, the stigma associated with being targeted or treated as a suspect can exacerbate feelings of alienation and distress, thereby worsening mental health outcomes (Kyprianides & Bradford, 2025).

The increasing demand on police forces to respond to mental health-related incidents is an international phenomena (Marcus & Stergiopoulos, 2022). It reflects both the inability of mental health services to manage an upsurge in demand, and changes in the way in which people present to public services in mental health emergencies. The response in England and Wales is to withdraw officers from responding to calls relating to welfare checks or people missing from mental health inpatient facilities (Home Office & Department of Health and Social Care, 2024b). While this may reduce some demand (Home Office & Department of Health and Social Care, 2024a), it does not address underlying issues such as disproportionate outcomes of police involvement for people from Black ethnic groups or the stigma which people with mental health problems experience when police officers are involved. Without a whole-system orientation tackling structural as well as operational challenges, reforms risk simply shifting responsibility between agencies rather than transforming the conditions that generate mental health problems in the first place. Therefore, enhanced training of police is a limited solution to the social and structural drivers of mental ill health among people and the communities within which they live (Kane et al., 2018; Scantlebury et al., 2017).

Some of the included studies had methodological limitations, including small samples and low response rates, which indicate that their findings must be treated with caution. Most of the included studies were conducted in the US, Canada or UK. More research is needed from other countries to observe policing in more diverse contexts. Additionally, more research of routine police work with people with mental health problems would also enhance the evidence base and provide some indications how responses to mental distress could potentially be improved so that fewer coercive interventions are required. This is particularly important as evidence suggests that negative interactions with the police are likely to further increase mental health problems (Jones & Thomas, 2019).

### Limitations

This review has focused on the policing of mental health problems outside of co-response models. Applying this eligibility criteria was occasionally problematic as it was not always clear if papers were reporting on policing models which involved mental health services or paramedics. Over 7,000 studies were screened for inclusion in the study and it is possible that some that were screened out included potentially useful data. However, the strict application of the eligibility criteria has helped us to reach some conclusions about the extent of evidence in this field.

Mental health is often invisible, both in terms of its presence in an incident involving the police and in police recording systems. There are also diverse ways to understand and define mental health. Also, police officers respond to what they are presented with and an individual’s mental health may impact on their behaviour, unbeknownst to the police. The impact of this is that mental health may remain invisible to researchers, making it difficult to review the full extent of policing skills and practices in this field. The included papers are likely to present data on the more apparent responses to people with mental health problems, which provides us with at least a glimpse into some of the issues involved in routine policing and mental health.

The review included a large number of papers and we used thematic synthesis to collate themes emerging from the findings of this body of literature. We used a rigorous process to develop the coding framework, though it is possible that this was influenced by researchers’ interests and perceptions of the themes. The studies were methodologically diverse, with a variety of strengths and weaknesses, and focused on a wide range of relevant topics. It is possible that some nuances were missed in the synthesis of data from the included papers, though it is hoped that the thematic analysis conveys the dominant findings and highlights gaps in the research in this field as intended.

## Conclusions

There is a widely held perception that attending to mental health calls is becoming an increasing part of routine policing. There is a concern that the police are not the most appropriate agency to respond to someone in emotional distress, but with increasing constraints on mental health care, they (and the ambulance service) are the only agencies able to make a rapid response (especially “out of hours”). Police value being supported by someone with mental health expertise especially in relation to decision making around risk. Police do possess appropriate psychosocial skills to engage with and de-escalate situations, and people and their carers report positive and compassionate responses. However, time pressures are a significant barrier to using such skills which can lead to the over-use of restrictive practices. There is a role for the police in certain circumstances, but police generally are not the most appropriate agency to be a first (or only response) to mental health concerns. Future research should address the role of the police in relation to non-mental health related contact with people with mental health problems (such as witnesses or victims of crime); the intersection of additional factors such as race, gender, housing status, substance use and how this impacts on police response and wider agency support and access; and how police, mental health and other agencies can improve communication, mutual understanding of roles and foster more positive cooperation. Future research should also seek to include the views of people with lived experience.

## Data Availability

No datasets were generated or analysed during the current study.
